# Differential genotypes of TNF-α and IL-10 for immunological diagnosis in discoid lupus erythematosus and oral lichen planus: A narrative review

**DOI:** 10.3389/fimmu.2022.967281

**Published:** 2022-08-05

**Authors:** Ruochong Wang, Xuefeng Zhang, Siyu Wang

**Affiliations:** ^1^ Emergency Department, State Key Laboratory of Oral Diseases, National Center of Stomatology, National Clinical Research Center for Oral Diseases, West China Hospital of Stomatology, Sichuan University, Chengdu, China; ^2^ Department of Dermatology, Institute of Dermatology and Venereology, Sichuan Academy of Medical Sciences & Sichuan Provincial People’s Hospital, Chengdu, China

**Keywords:** discoid lupus erythematosus, oral lichen planus, TNF-α, IL-10, immune homeostasis

## Abstract

Discoid lupus erythematosus and oral lichen planus are chronic systemic immune system-mediated diseases with unclear etiology and pathogenesis. The oral mucosa is the common primary site of pathogenesis in both, whereby innate and adaptive immunity and inflammation play crucial roles. The clinical manifestations of discoid lupus erythematosus on the oral mucosa are very similar to those of oral lichen planus; therefore, its oral lesion is classified under oral lichenoid lesions. In practice, the differential diagnosis of discoid lupus erythematosus and oral lichen planus has always relied on the clinical manifestations, with histopathological examination as an auxiliary diagnostic tool. However, the close resemblance of the clinical manifestations and histopathology proves challenging for accurate differential diagnosis and further treatment. In most cases, dentists and pathologists fail to distinguish between the conditions during the early stages of the lesions. It should be noted that both are considered to be precancerous conditions, highlighting the significance of early diagnosis and treatment. In the context of unknown etiology and pathogenesis, we suggest a serological and genetic diagnostic method based on TNF-α and IL-10. These are the two most common cytokines produced by the innate and adaptive immune systems and they play a fundamental role in maintaining immune homeostasis and modulating inflammation. The prominent variability in their expression levels and gene polymorphism typing in different lesions compensates for the low specificity of current conventional diagnostic protocols. This new diagnostic scheme, starting from the immunity and inflammation of the oral mucosa, enables simultaneous comparison of discoid lupus erythematosus and oral lichen planus. With relevant supportive evidence, this information can enhance physicians’ understanding of the two diseases, contribute to precision medicine, and aid in prevention of precancerous conditions.

## Introduction

Lupus erythematosus is a general term for a group of chronic, recurrent autoimmune diseases that manifests in two main forms: systemic lupus erythematosus (SLE), which involves multiple organs, and cutaneous lupus erythematosus (CLE), which is limited to the skin and mucosae (1). Among CLEs, discoid cutaneous lupus erythematosus (DLE) is most likely to invade the oral mucosa, and the ensuing oral lesions are classified as oral lichenoid lesions (OLLs) (2), due to the close clinical resemblance to oral lichen planus (OLP), characterized by features such as plaques, petechiae, and oral erosions/ulcers. On further histological examination, both DLE and OLP exhibit incomplete epithelial keratinization, subepithelial lymphocytic infiltration, and the presence of cytoid bodies in the granular and keratinized layers (3). Owing to the tremendous similarities between DLE and OLP, clinicians experience considerable difficulty in differential diagnosis. Researchers (3, 4) reported that differences in clinical manifestation are almost nonexistent and reliable diagnostic tests are unavailable. Patients with similar symptoms of lichen planus should be referred to hospital for biopsy or other tests to confirm the diagnosis (2). However, it should be noted that both DLE and OLP possess the potential for malignant transformation, and biopsy of the lesion area could accelerate this process by stimulating precancerous lesions (5, 6). Therefore, a minimally invasive diagnostic method is urgently needed.

Despite the unclear etiology, it is known that DLE and OLP are regulated by the immune system. Multiple immune factors participate in the pathogenesis of DLE and OLP. Therefore, to address this clinical dilemma, it is necessary to identify immune factors that are strongly correlated with DLE and OLP, and to achieve a definite diagnosis by detecting differences among them. During our investigation, we found that a series of prospective studies and reviews highlight genotypes and expression levels of IL10 and TNF-alpha as characteristic and promising immunologic differentiators. It is expected that more accurate diagnosis will facilitate more precise and timely treatment.

## Challenges in identification

OLP is a common chronic inflammatory disease that manifests with oral mucosal and cutaneous lesions. It is characterized by lacelike white stripes on the oral mucosa with an abnormality of keratinization. The distribution of the lesions is primarily bilateral and symmetrical, particularly in the buccal mucosa. The disease can be classified as reticular, plaque-like, atrophic, bullous, erosive, and popular types according to their morphological characteristics. During the clinical course of the disease, the different types of lesions can transform into each other. After remission of the disease, pigmentation may remain on the mucosa. The pathological hallmarks are liquefied degeneration of the basal lamina and dense lymphocytic infiltration of the lamina propria with a predominance of T cells. OLLs are a heterogeneous group of diseases with clinical manifestations and histopathology similar to OLP, also known as oral lichenoid reactions (OLRs). In 2006, at the World Workshop in Oral Medicine IV, OLLs were separated into oral lichenoid contact lesions (OLCL) caused by contact with dental restorative materials, oral lichenoid drug reactions (OLDRs) caused by certain systemic drugs, and oral lichenoid lesions of graft-vs.-host disease (GVHD) (7). The WHO then elucidated (8) the features of OLL and OLP in 2007, but the diagnosis of OLL was based on exclusion rather than inclusion. Therefore, the WHO classification was only a descriptive list rather than a clinical guideline. In 2019, researchers (4) concluded that these classifications were outdated and incomplete, and failed to provide clear and reliable clinical and histological criteria; the classification of OLL was eventually expanded to thirteen clinical entities, that included DLE and other disorders. Nevertheless, Lu et al. (9) recently highlighted that to date, a widely acknowledged and accepted OLL classification was still lacking.

Progress regarding the development of OLL classifications has been achieved by the inclusion of more subtypes and the addition of OLL definitions. At present, DLE is classified as a type of OLL because of similarities in clinical and histopathological manifestations, cell kinetics, immune alterations, treatment, and prognosis. First, from an epidemiological perspective, young and middle-aged women are susceptible to both disorders. In terms of clinical manifestations, oral lesions of DLE occur on the buccal mucosa, vermilion, gingiva, dorsal tongue, ventral tongue and palate; whereas for OLP, any part of the oral mucosa could be affected, with the buccal mucosa being the most common (10). Oral lesions of DLE range from dark red papules to well-circumscribed white lacy plaques with hyperkeratosis, and those with central ulceration possess radiolucent fine and short white lines. A characteristic of DLE lesions is that the lesion area can extend onto the skin beyond the vermilion margin, which can be observed in the early stages. However, after further erosion of the ulcerated surface, it is obscured by the formation of a crust resulting from hemorrhage combined with infection. DLE lesions heal with scarring, atrophy, and peripheral hyper- or hypopigmentation. OLP manifests as small papules in a linear white pattern with abnormal keratinization. The surrounding mucosa around the white lesions may become congested, ulcerated, and eroded. Pigmentation is retained on the mucosa after regression. A recent comparative analysis (11) revealed that red macules, telangiectasia, and discoid plaques were more common in oral lupus erythematosus (OLE), whereas reticulated patches were more typical in OLP. Nevertheless, the authors acknowledged that despite this, significant overlap remains between OLE and OLP. The greatest difference in clinical manifestation between DLE and OLP may lie in the fact that the former is mainly unilateral and asymmetric in distribution, but the latter is primarily bilateral and symmetrical (4). However, this evidence is insufficient to support the diagnosis, and the diseases still appear similar, if not identical, in most cases; diagnosis currently relies on laboratory tests. Previous studies demonstrated that histopathological differentiation between DLE and OLP is in most cases equivocal or non-specific (12). Both present as epithelial dyskeratosis with keratin plugging; inflammation in the lamina propria can be a mixed inflammatory cell infiltrate or a lymphocyte-rich infiltrate, ranging from paucicellular to band-like; basal cells are liquefied and degenerated, and the basement membrane is indistinct (13) (**Table 1**).

**Table 1:** Classification and characteristics of OLP and DLE.

Diseases	DLE	OLP
Prevalence	Young and middle-aged women	
Oral mucosal lesions	Unilateral affecting mainly on the hps. may exceed the vermilion and reach the skin; erythema or erosion surrounded by radiolucent fine and short white lines	Bilateral affecting mainly on the buccal area; irregularly shaped pearly white stripes or plaque with or without erosion and ulceration
Cutaneous lesions	Localized to head, face, auricles and sun-exposed areas: telangiectasia; hypopigmentation; butterfly-shaped arythema on the face	Bilateral affecting flexors of the extremities; light purle polygonal flat papules with waxy sheen
Histopathological features	Incomplete keratinizantion of the epithelium: prominently hyperplasia of the prickle cell layer; liquefied degeneration of the basal lamina; scattered infiltration of lymphocytes of the lamina propria; dense lymphocytic infiltration around the blood vessels, predominantly T cells	Incomplete keratinization of the epithelium; atrophy of the prickle cell layer; liquefied degeneration of the basal lamina; dense lymphocytic infiltration of the lamina propria with a predominance of T cells; little lymphocytic infiltration around the blood vessels
Immunopathological features	DIF: linear emerald green flourescent band or continuous IgG, IgM, IgA, C3 and fibrinogen deposition at BMZ, with or without IgM, IgG-positive cytiods bodies IIF: negative	DIF: granular or shaggy deposit of fibrin, fibrinogen, immunoglobulin, and C3 at BMZ. With or wothout IgM-positive cytoid bodies IIF: negative
Prognosis	Precancerous conditions	

OLP, oral lichen planus; DLE, discoid lupus erythematosus; DIF, direct immunofluorescence; IIF: indirect immunofluorescence; BMZ, basement membrane zone

Despite consideration of the clinical details, pathologists can only conservatively report the lesion as either OLP or OLL (2). Lu et al. (9) suggested that the term OLL should only be used for provisional diagnosis, and the specific subtype of OLL should be clearly indicated in the final diagnosis. Therefore, given the predicament of histopathology, the current research explores the potential of immunopathological examination and genetic testing.

## Opportunity for differential diagnosis

Immunological methods have been widely used as an adjunctive diagnostic tool for OLP and DLE. Unlike pemphigus, pemphigoid, and other oral mucosal autoimmune diseases, DLE and OLP lack specific pathological manifestations. The immunopathological diagnosis mainly relies on observing the distribution and types of various immune factors. In an earlier comparative study (14), it was found that the diagnostic specificity of the immunofluorescence (IF) technique was greater than that of histopathology in both DLE and OLP, and the most discriminatory immunohistochemical features between DLE and OLP were the incidence and morphological pattern of IgG along the epidermal basement membrane. Subsequently, in cases of DLE, a continuous, thick and thin emerald green fluorescent band at the basement membrane zone (BMZ) was observed using direct immunofluorescence (DIF). In addition to IgG, IgM and C3 were deposited in a granular or shaggy pattern, known as a lupus band (15, 16). Moreover, IgM is considered the most commonly identified immunoreactant in OLE, whereas C3 is more common in other oral lesions (17, 18). Further, statistics show that DIF is positive in approximately 70% of DLE tissue samples, whereas indirect immunofluorescence (IIF) is generally negative and not recommended for the diagnosis of DLE or OLP (13, 19). In OLP cases, DIF displayed granular or shaggy deposits of fibrin, fibrinogen, immunoglobulin, and C3 along the BMZ (14, 20). Although most researchers agreed that the presence of the lupus band observed by DIF would help confirm the diagnosis, Carrozzo et al. (21) contended that the lupus band is neither sufficiently sensitive nor specific to be a reliable diagnostic method. Despite its characteristics, it fails to be pathognomonic of DLE (22). The combination of serological biomarkers and immunohistochemical tests is expected to replace the flawed lupus band test. Unfortunately, since the etiology and pathogenesis remain unclear, no prominent autoantibodies or antigens to OLP or DLE are available for detection in the serum. Current research is directed at exploring some of the marked distinctions among inflammatory molecules typical of the disease.

Heat shock proteins (HSPs) have been implicated as antigenic stimuli of autoimmune diseases, also known as “stress proteins” and “molecular chaperones”. The two subtypes of HSPs, HSP60 and HSP70, are highly competent for T-cell activation, and histological studies demonstrate that OLP is a T-cell mediated disease (23, 24). Meanwhile, multiple case studies have observed significantly higher serum levels as well as mRNA overexpression of HSP60 and HSP70 in OLP cases compared to those in healthy controls (25–27). However, other studies revealed no statistically significant differences in HSP70 expression between OLP and normal mucosa (28). The expression of HSP60 is prominent in OLP, but it is not specific and can be seen in other lesions (29). Therefore, Mohtasham et al. (27) noted that upregulation of HSPs is not yet qualified as a diagnostic tool, and immunohistochemical tests or quantitative evaluations of HSPs for all cases of OLP are not recommended. Both the lupus band test and the quantitative evaluations of HSPs are only applicable to one of them, thus automatically excluding the other. A method that allows simultaneous comparison of the two diseases would be more specific and more rigorous.

### TNF-α in OLP

TNF-α belongs to the TNF/TNFR cytokine superfamily, and is one of the most important pro-inflammatory cytokines and potent immunomodulators that regulates a wide range of immune-related activities, including inflammation, innate and adaptive immune responses, and autoimmunity (30). TNF-α is secreted by a variety of cells, such as activated monocytes, macrophages, B cells, T cells, mast cells, and fibroblasts (31). The histological evidence has proved that there is increased T-lymphocytic infiltration in OLP. Accordingly, case studies have shown that TNF-α was upregulated in lesional T cells and serum from OLP patients, and lesional T cells contain TNF-α mRNA and express TNF-α cytokines (32–35). In addition to the role of TNF-α produced by T cells, TNF-α is capable of enhancing TCR-dependent activation of CD8+ cytotoxic T cells (36). Histological studies further revealed that most OLP-associated T cells are activated CD8+ cytotoxic T cells (37). This is consistent with the cellular mechanism whereby CD8+ cytotoxic T cells trigger apoptosis of keratinocytes in OLP lesions by releasing TNF-α to bind to TNF-α receptor 1 (TNFR1) on the surface of keratinocytes (38).

The gene for TNF-α is located on chromosome 6q21, which is a highly polymorphic region (39). TNF-α possesses a large number of polymorphisms, reflected by having up to 14 alleles. Those alleles differ from their biallelic single nucleotide polymorphisms (SNPs), which are at different positions relative to the transcription start site and are mainly G to A substitutions. The most investigated polymorphisms in the promoter region of the TNF-α gene are those at positions-308 (rs1800629) and -238 (rs361525) (40). The A allele of SNP-308 promotes TNF-α expression, which is a stronger transcriptional activator than the G allele after *in vitro* lymphocyte stimulation (41, 42). A meta-analysis revealed that the TNFα-308 G/A polymorphism was a potential genetic marker for OLP (43). The TNF-α gene polymorphisms vary not only between individuals, but also between populations. Bai et al. (44) first demonstrated that the frequencies of the TNF-α-308A allele in patients with erosive OLP was significantly higher than that in controls in a Chinese Han cohort. Subsequently, a higher proportion of OLP patients with the TNF-α-308 AA genotype (high producer genotype) than with the other genotypes was found in the Thai population; also, the TNF-α-308 AA genotype was associated with an increased risk of developing erosive OLP. The association between the TNF-α promoter region at positions -863 and -238 and the disease was excluded (45). A close relationship between allele A of TNF-α (-308G/A) and OLP was also established in the Arabian OLP community (46). Several genetic studies have suggested a positive relationship between OLP and elevated TNF-α from the incidence in different races, which may be a certain epidemiological pattern. Genetic studies are also consistent with histological studies. Since the TNF-α-308 AA genotype is more predominant in erosive OLP and more keratinocyte apoptosis is observed in patients with erosive OLP, it can be inferred that the TNF-α-308 AA genotype leads to a higher production of TNF-α, which promotes CD8+ cytotoxic T cell-induced apoptosis in keratinocytes in OLP lesions (47).

### TNF-α in DLE

On the contrary, Werth et al. found that polymorphism of TNF-α promoter -308A is not associated with DLE (48). In a large genetic study, using allele-specific probes and real time RT-PCR, researchers concluded that the high TNF-α producer group (-308AA or AG) was associated with SLE, while in the low TNF-α producer group (-308GG), the risk and prevalence of DLE was higher (49). Another difference from OLP is that gene expression microarrays and miRNA screenings showed an enrichment of CD4+ T cells in DLE lesions rather than CD8+ T cells that primarily promote TNF-α production (50). From a therapeutic perspective, thalidomide, an effective treatment for DLE, has long been believed to exert its anti-inflammatory effects by targeting the 3’-untranslated region (3’-UTR) of TNF-α mRNA and inhibiting TNF-α production by monocytes (51, 52). This concept is challenged by the above genetic research in addition to a recent review on the efficacy of thalidomide on DLE, which stated that thalidomide could not be considered to treat DLE by inhibiting TNF-α because of conflicting results from some studies (53). Furthermore, several case reports showed that skin symptoms suspected to be DLE or CLE developed after application of infliximab, adalimumab and bevacizumab (TNF-α inhibitors), indicating that TNF-α inhibitors may not be appropriate for the treatment of DLE (54–56). Therefore, the theory that thalidomide acts by inhibiting TNF-α production is questionable, and its anti-inflammatory effect may be exerted by modulating other inflammatory cytokines. In conclusion, differences in TNF-α levels and polymorphisms are a major difference between OLP and DLE. The isolated assessment of the sole cytokine, though of relevance, does not provide a comprehensive understanding of the impact of inflammation on the disease. To design a more sensitive diagnostic modality, other cytokines with significant differences also need to be identified.

### IL-10 in DLE

IL-10 gene maps to the junction of 1q31-q32. Similar to TNF-α, the IL-10 gene promoter is strongly polymorphic. In addition to the two microsatellites, three SNPs located at positions 1082 (G/A) (rs18000896), 819 (C/T) (rs1800871) and 592 (C/A) (rs1800872) generate three haplotypes (GCC, ACC and ATA), which are associated with the transcription rate of IL-10 and variability in IL-10 production (57, 58). IL-10 production levels are elevated in the GCC haplotype, relatively intermediate in the ACC haplotype, and low in the ATA haplotype (57, 59). Patients with the GG genotype at the -1082 position are referred to as the high IL10 producer group, due to the association with high IL-10 transcript levels (49).First, it can be inferred from the low level of TNF-α in DLE that the level of IL-10 expression may be high. It has been reported that IL-10 was constitutively expressed in keratinocytes; its expression was augmented by ultraviolet exposure (60). Continuous irradiation did not only lead to IL-10 stimulation of dermal endothelial cells to produce pro-inflammatory cytokines and chemokines, but also acted as a risk factor for DLE, which linked high IL-10 production to DLE (61). As previously described, gene expression profiling techniques in DLE displayed CD4+ T cell-enrichment in which the Th1 response was predominant. Although IL-10 suppresses Th1 cells, Th1 cells are its main source in adaptive immunity (62). As Th1 cells dominate, IL-10 secretion surges. However, local overexpression of IL-10 disrupted the balance and induced inflammation instead of serving as an anti-inflammatory agent. After suggesting the relationship and indivisibility of TNF-α and IL-10, Suárez et al. (49) then launched a genotypic analysis of IL-10 using the same approach as the evaluation of TNF-α and concluded that the highest risk of developing DLE was found in individuals with a combined high IL10/low TNF-α genotype, which more likely resulted from overexpression of IL-10 rather than low production of TNF-α. In addition, cytokine interactions and the presence of a high IL-10/low TNF-α genotype suggest once again that the target of thalidomide for DLE is not TNF-α and that other TNF-α inhibitors are unsuitable for the treatment of DLE. Another study highlighted that thalidomide inhibited regulatory T cell (Treg) activity, which is a cell type in CD4+ T cell subsets and a source of IL-10 (63). A reduction of Treg response in DLE lesions would reduce IL-10 production. This may be the potential pharmacological mechanism of thalidomide in DLE.

### IL-10 in OLP

**Figure 1:**
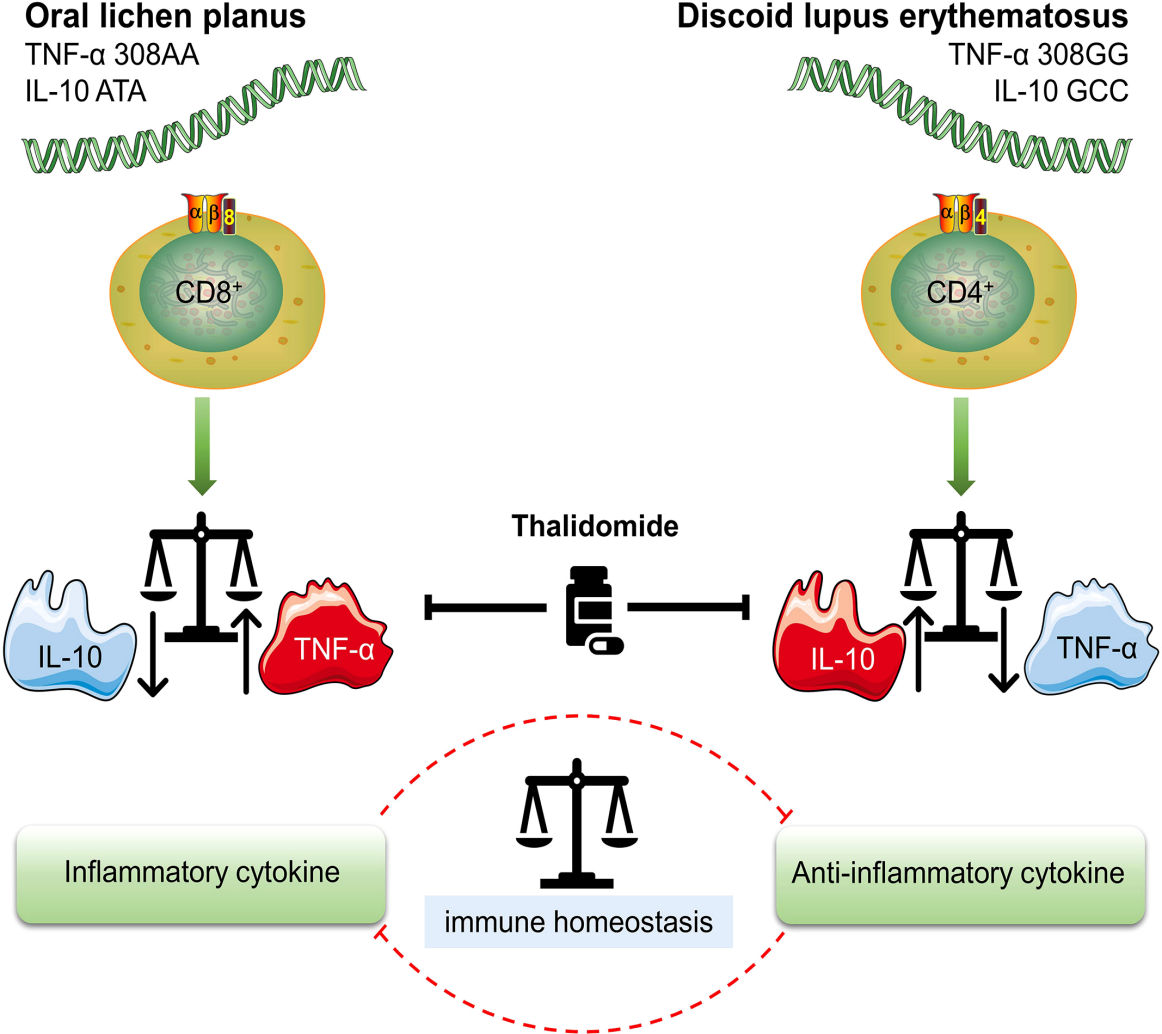
Different genotypes of OLP (oral lichen planus) and DLE (discoid lupus erythematosus) High TNF-α (TNF-α 308AA)/low IL-10 (IL-10 ATA) genotype is characterized by a higher production of TNF-α and is more likely to result in OLP. TNF-α is mainly secreted by CD8+ cytotoxic T cells, which are also the most OLP-associated T cells. High IL-10 (IL-10 GCC)/low TNF-α (TNF-α 308GG) genotype suggests increased risk and prevalence of DLE. The overexpression of IL-10 is associated with the enrichment of CD4+ T cell subsets. Treg (regulatory T cell) and Th1 (T helper 1 cell), two typical CD4+ T cell subsets, are sources of IL-10 in DLE. Either an increase or decrease in TNF-α and IL-10 leads to an imbalance in the immune system, which ultimately initiates the onset of disease. Thalidomide is a multi-targeted IMiD (immunomodulatory drug) that inhibits both TNF-α and IL-10. The inhibition of IL-10 may be the underlying pharmacological mechanism in the treatment of DLE.

An *in vitro* analysis of IL-10 mRNA and expression using 35S-labelled oligonucleotide probes and polymerase chain reaction showed that cells capable of generating IL-10 mRNA were present in the original lesions of OLP (32). Gene polymorphisms for both TNF-α and IL-10 were included in a survey of a Chinese Han population by Bai et al. The results for TNF-α were as described previously, while similar results to other genetic studies were found for IL-10: the ATA haplotype was correlated with a low serum level of IL-10. More importantly, they identified a possible association between the ATA haplotype and OLP (44). Not coincidentally, haplotype ATA extracted from the 1082G/A, -819C/T, and -592C/A polymorphisms of the IL-10 gene were likewise found to be more prevalent in patients with OLP in the Arab population, with similar findings as in the Han population (46). However, this study was not conducted in the Thai population. Lu et al. demonstrated in their latest work that there was a tendency for decreased serum IL-10 levels in patients with OLP (64). All the above suggest that serum IL-10 levels are low in OLP (**Figure 1**).

### The association between TNF-α and IL-10

Inflammatory cytokines do not act independently, but in a cytokine network. Suárez et al. (49) suggested that the role of cytokines may be profoundly constrained by the presence of other cytokines, particularly in the case of TNF-α and interleukin-10, which have complex and mainly opposing roles. IL-10 is a multi-functional cytokine with prominently anti-inflammatory effects, that inhibits the synthesis of many other cytokines, notably TNF-α (65). Its mechanism is to limit collateral damage to host cells and tissues during the inflammatory response and to maintain the balance between the inflammatory and anti-inflammatory responses (66). It is considered to be an important factor in peripheral tolerance and a major suppressor of inflammation; it is involved in the inactivation of monocytes and macrophages, inhibition of T helper 1 (Th1) cells and promotion of B-cell proliferation and differentiation (67). The most significant of these is the inactivation of monocytes and macrophages, thereby inhibiting TNF-α release from these cells (66). Moreover, to maintain the balance, IL-10 expression can in turn be attenuated or compromised by the cytokines produced by these cells (68). For example, TNF-α modulated the differentiation state and expression of IL-10 in human CD4+ T cell subsets, which was proven by IL-10 production enhancement after applying therapeutic antibodies blocking TNF-α (69).

## Other indexes and methods for further diagnosis of OLP

Compared to DLE, OLP has received more research attention. In recent years, diagnostic indexes with satisfactory sensitivity and specificity have been frequently reported. OLP is a well-defined precancerous condition. The less irritation to the lesion area, the better the control of disease progression. Therefore, alternative diagnostic methods should avoid direct irritation of the lesion area. In order to achieve a minimally invasive to non-invasive solution, whole saliva can be collected and screened for salivary TNF-α levels by ELISA. There are documented statistics of higher levels of TNF-α in saliva compared to that in the serum in patients with OLP, suggesting that measuring this biomarker in saliva not only does not provoke precancerous lesions but may also be more prominent than in serum (70). Another typical pro-inflammatory cytokine, IL-17, has been found to have a diagnostic role similar to that of TNF-α in the serum and saliva of OLP patients (71, 72). Other researchers have also used saliva samples to identify fibrinogen fragment D, complement component C3c, and cystatin SA as putative biomarkers for the screening and diagnosis of OLP (73). In the recent literature, researchers (74, 75) first concluded that there is an association between serum/saliva levels of multiple pro-inflammatory cytokines and OLP pathogenesis, and then suggested the use of saliva and serum C-Reactive Protein (CRP) and total antioxidant capacity (TAC) in the assessment of OLP development.

In addition, if the diagnosis of OLP is accomplished after the exclusion of DLE, the HSP test can be reintroduced at this time to corroborate the genetic test result of TNF-α for further evaluation of the disease. The HSP test is mainly valuable in determining the prognosis of the disease and for use in treatment planning. Because HSP60 expression and HSP70 expression were increased in erosive and atrophic subtypes of OLP, which are more prone to carcinogenesis, they are known as the “fingerprints” of a generalized immune response in immune-mediated diseases (27). Therefore, screening of serum levels of HSP60 and HSP70 aids further differentiation and detection of erosive OLP and non-erosive OLP (**Table 2**).

**Table 2:** Samples and assay indexes for differential diagnosis.

	Peripheral blood		Whole saliva	
Disease	DLE	OLP	DLE	OLP
TNF-α	↓	↑	\	↑
IL-10	↑	↓	\	\
allele A of TNF-α (-308 A>G) (rs1800629)	\	↑	\	\
allele G of IL-i0 (1082 A>G) (rs18000896)	↑	\	\	\
IL-17	\	↑	\	↑
fibrinogen fragment D	\	\	\	↑
Complement component C3c	\	\	\	↑
cystatin SA	\	\	\	↓
TAC	\	↓	\	↓
CRP	\	↑	\	↑
HSP60	\	↑	\	\
HSP70	\	↑	\	\

↓, down-regulated; ↑, up-regulated; \, no available.

## Discussion

Both OLP and DLE have been identified as precancerous conditions (76, 77). The risk of malignant transformation of OLP to oral squamous cell carcinoma has been estimated to be 1– 2% (5). In the recent international consensus report on nomenclature and classification of oral potentially malignant disorders (OPMDs), DLE was also described as an independent disorder in the categories (78). Their malignant potential places high emphasis on early diagnosis and treatment. However, the specific genes responsible for OLP or DLE have not yet been identified. Although some studies are probing for genetic susceptibility and biomarkers using bioinformatic approaches, only an approximate range of candidate gene regions and differentially expressed genes have been identified, which cannot yet be applied in clinical practice (79, 80).

Since the clinical manifestations and histopathological features of both diseases are extremely similar, attention has focused on immunological exploration in recent years. Pathologists have adopted DIF as an adjunctive diagnostic tool. Sun et al. (81) suggested that DIF on fresh frozen tissue from the lesion site of OLP or DLE was preferred. The presence of DLE can be determined by observing the lupus band. However, the reliability of the lupus band test is being questioned. It has been found that patients with DLE scarcely fulfill four or more criteria for SLE, with the lupus band test as one of the criteria (82). Nowadays, serologic testing has achieved great progress. The advantages lie in the rapid results and the clear indication of further treatment. Immunohistochemistry of HSPs in OLP is a potential test option, but some researchers question its practicability (27). Again, there is no evidence that serum and mRNA levels of HSPs in DLE show similarly significant changes as observed in OLP. On the other hand, studies at the molecular level have enabled understanding of inflammation to surpass the limitations of type discrimination among inflammatory factors, initiating tools that reflect the nature of inflammation for clinical diagnosis and epidemiological studies.

**Figure 2:**
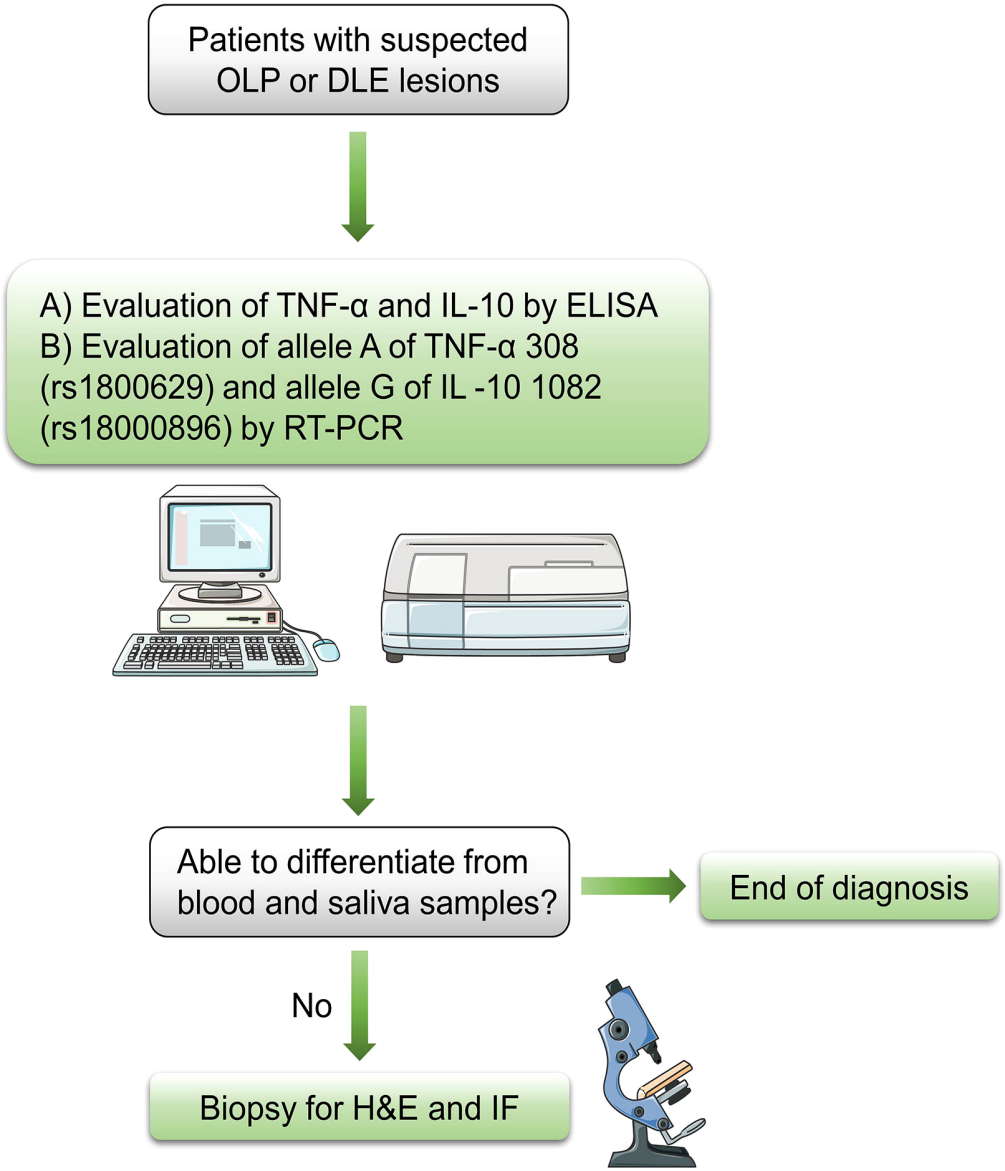
Flow chart for diagnosis of suspected OLP or DLE lesions OLP, oral lichen planus; DLE, discoid lupus erythematosus; ELISA, enzyme linked immunosorbent assay; RT-PCR, reverse transcriptase polymerase chain reaction; H&E, hematoxylin and eosin; IF, immunofluorescence.

Because they modulate inflammatory and anti-inflammatory responses, TNF-α and IL-10 play pivotal roles in the pathogenesis of DLE and OLP, both diseases mediated by the immune system. Moreover, because of the simultaneous comparison of the two diseases, which is superior to the characteristic lupus band test for DLE and the HSP test for OLP, the two cytokines also exhibit considerable potential for differential diagnosis. We believe that the serum level of both cytokines can be evaluated qualitatively by conventional methods such as immunofluorescence and enzyme linked immunosorbent assay (ELISA), and then the frequency of their alleles can be determined qualitatively by typing gene polymorphisms using a polymerase chain reaction (PCR) with sequence specific primers, i.e., allele A of TNF-α (-308 A>G) and allele G of IL -10 (-1082 A>G). This novel diagnostic scheme is expected to facilitate the screening of DLE from the previous histopathological classification of OLLs or, more directly, to confirm suspected cases as DLE or OLP. Therefore, the protocol for the admission of patients is described as below: the initial step is the exclusion of other definable diseases or impairments based on evidence from the initial clinical examination, followed by the above recommended methods for ancillary diagnosis. Given the invasive nature of surgical biopsy and the potentially malignant nature of OLP and DLE, the lesional biopsy is the last diagnostic option to be considered (**Figure 2**).

Although the findings of this study have a number of important implications for future practice, the generalizability of the novel diagnostic scheme is subject to certain limitations. The expression patterns of inflammatory factors may be extremely diverse due to the induction of other inflammatory mediators and individual genetic predisposition. For instance, it is doubtful whether serum levels of IL-10 are reduced in patients with OLP, since two studies observed elevated serum levels of IL-10 in patients with OLP a decade ago (83, 84). It was also reported that serum levels of IL-10 were lower in patients with DLE than healthy people (85). Therefore, more accurate data from laboratory tests and more extensive evidence from evidence-based medicine are required to assist in the establishment of greater accuracy of the diagnostic scheme in the future. Until the real pathogenesis of the two diseases is ascertained, research targeting TNF-α and IL-10 will not only facilitate the discovery of the pathogenesis of DLE and OLP, but also serve as an alternative and superior diagnostic option.

## Author contributions

XFZ and SYW: Administrative, technical, and material support; study supervision; data acquisition; conceptualization and design of the manuscript. RCW, XFZ and SYW: Manuscript writing, reviewing, and revision. All authors approved the final manuscript and agreed to be accountable for all aspects of the work. Furthermore, all authors ensured that questions related to the accuracy or integrity of any part of the work were appropriately investigated and resolved.

## Funding

The National Natural Science Foundation of China (NSFC) (Grant Nos. 82002877) and Young Talent Project of Sichuan Provincial People’s Hospital (No. 2016QN15) contributed to the design of the study, the collection, analysis, and interpretation of data, and writing the manuscript.

## Conflict of interest

The authors declare that the research was conducted in the absence of any commercial or financial relationships that could be construed as a potential conflict of interest.

## Publisher’s note

All claims expressed in this article are solely those of the authors and do not necessarily represent those of their affiliated organizations, or those of the publisher, the editors and the reviewers. Any product that may be evaluated in this article, or claim that may be made by its manufacturer, is not guaranteed or endorsed by the publisher.
